# The potential of microRNAs as human prostate cancer biomarkers: A meta‐analysis of related studies

**DOI:** 10.1002/jcb.26445

**Published:** 2017-12-04

**Authors:** Chun‐Jiao Song, Huan Chen, Li‐Zhong Chen, Guo‐Mei Ru, Jian‐Jun Guo, Qian‐Nan Ding

**Affiliations:** ^1^ Medical Research Center Shaoxing people's Hospital, Shaoxing Hospital of Zhejiang University Shaoxing China; ^2^ Zhejiang Institute of Microbiology Key Laboratory of Microorganism Technology and Bioinformatics Research of Zhejiang Province Hangzhou China

**Keywords:** biomarker, meta‐analysis, microRNA, prostate cancer

## Abstract

Prostate cancer (PC) is a very important kind of male malignancies. When PC evolves into a stage of hormone resistance or metastasis, the fatality rate is very high. Currently, discoveries and advances in miRNAs as biomarkers have opened the potential for the diagnosis of PC, especially early diagnosis. miRNAs not only can noninvasively or minimally invasively identify PC, but also can provide the data for optimization and personalization of therapy. Moreover, miRNAs have been shown to play an important role to predict prognosis of PC. The purpose of this meta‐analysis is to integrate the currently published expression profile data of miRNAs in PC, and evaluate the value of miRNAs as biomarkers for PC. All of relevant records were selected via electronic databases: Pubmed, Embase, Cochrane, and CNKI based on the assessment of title, abstract, and full text. we extracted mean ± SD or fold change of miRNAs expression levels in PC versus BPH or normal controls. Pooled hazard ratios (HRs) with 95% confidence intervals (CI) for overall survival (OS) and recurrence‐free survival (RFS), were also calculated to detect the relationship between high miRNAs expression and PC prognosis. Selected 104 articles were published in 2007‐2017. According to the inclusion criteria, 104 records were included for this meta‐analysis. The pooled or stratified analyze showed 10 up‐regulated miRNAs (miR‐18a, miR‐34a, miR‐106b, miR‐141, miR‐182, miR‐183, miR‐200a/b, miR‐301a, and miR‐375) and 14 down‐regulated miRNAs (miR‐1, miR‐23b/27b, miR‐30c, miR‐99b, miR‐139‐5p, miR‐152, miR‐187, miR‐204, miR‐205, miR‐224, miR‐452, miR‐505, and let‐7c) had relatively good diagnostic and predictive potential to discriminate PC from BPH/normal controls. Furthermore, high expression of miR‐32 and low expression of let‐7c could be used to differentiate metastatic PC from local/primary PC. Additional interesting findings were that the expression profiles of five miRNAs (miR‐21, miR‐30c, miR‐129, miR‐145, and let‐7c) could predict poor RFS of PC, while the evaluation of miR‐375 was associated with worse OS. miRNAs are important regulators in PC progression. Our results indicate that miRNAs are suitable for predicting the different stages of PC. The detection of miRNAs is an effective way to control patient's prognosis and evaluate therapeutic efficacy. However, large‐scale detections based on common clinical guidelines are still necessary to further validate our conclusions, due to the bias induced by molecular heterogeneity and differences in study design and detection methods.

## 1 INTRODUCTION

Prostate cancer (PC) is the leading male cancer worldwide. In 2016, PC is estimated to be responsible for 26 120 deaths in the United States.^1^ Early PC is localized which can be curable by a variety of therapies: chemotherapy, radiation therapy, radical prostatectomy, and cryotherapy, etc. Unfortunately, approximately 23‐40% of these patients would go on to develop metastatic tumors after initial therapy.^2^ Prostate tumors often metastasize to bone and other organs to cause patients death.^3^ At present, metastatic cases are treated with androgen‐deprivation therapy to induce apoptosis of tumor cells or to inhibit cells growth. This will further induce PC to be insensitive to hormone and progress to CRPC, which is essentially untreatable.

In spite of the prevalence of PC, there are no diagnostic or prognostic biomarkers to specifically and precisely distinguish its aggressiveness. In the early 1990s, the detection of PC dramatically increased due to the introduction of the prostate‐specific antigen (PSA) test, which had been used as a routine assay in clinic. PSA levels are not specific for PC, and may fluctuate to induce false‐positive due to infections, inflammation, or hyperplasia, etc. Due to the poor correlation between PSA levels and PC which leads to overdiagnosis and overtreatment,^4^, ^5^ the US Preventive Services Task Force recommends physicians not to routinely perform PSA‐based screening.^6^, ^7^, ^8^ Moreover, the prostate needle biopsy has also obvious defects because only 2% of the prostate tumor samples can be sampled by puncture.^9^ Therefore, we still need to seek unique biomarkers discriminating different stages of PC.

miRNAs are small, single‐stranded, non‐coding, 21‐23 nucleotides RNAs that are conserved and endogenous, and have been shown to regulate the expression of approximately 60% of human genes.^10^ miRNAs post‐transcriptionally regulate gene expression via base‐pairing with 3′‐untranslated regions (UTRs) of mRNA, and are found to be located in fragile regions involved in various cancers.^11^ miRNAs may regulate a wide range of biological processes: proliferation, apoptosis, development, and differentiation, etc, and are discovered to be aberrantly expressed in various carcinomas. Thus, more and more researchers are willing to consider miRNAs as diagnostic or prognostic biomarkers. Recently, miRNAs have attracted the attention of urologists and oncologists, because of their potential uses for the urologic cancers diagnosis, monitoring, and treatment. Specific miRNA may be used as marker to detect PC, predict prognosis, and monitor therapy. miRNAs are attractive biomarkers because they can be easily extracted from a wide range of biologic samples, and are stable in various storage conditions. Furthermore, miRNAs can be accurately detected by a variety of techniques, for example, qRT‐PCR, microarray, and next‐generation sequencing, etc. However, there are some controversies on miRNAs as biomarkers, because some studies obtain statistically insignificant results, and some draw inconsistent conclusions. In view of these results from different patient cohorts or various detection methods or different data analysis platforms, miRNAs are still considered an attractive biomarkers to assess recurrence and therapeutic effect. Therefore, we conducted a meta‐analysis to clarify the role of miRNAs for tumor progression and RFS and OS in PC clinical specimens.

## 2 MATERIALS AND METHODS

### 2.1 Search strategy

We performed a detailed literature search in PubMed, Embase, Cochrane, and Chinese National Knowledge Infrastructure databases to obtain relevant articles for this meta‐analysis. Relevant studies were selected according to a combination of keywords and Medical Subject Headings (MeSH): (“prostate cancer” or “prostate neoplasm” or “prostate tumor”) and (“microRNAs” or “miRNAs” or “miR‐”) and (“marker or “biomarker”). All selected studies in English or Chinese were viewed, and their reference lists were also examined for other eligible publications. Most studies were published between 2007and 2017. The last search update was finished on July 8, 2017. These studies regarding miRNAs and PC are performed in clinical samples or PC cell lines. Published data are subject to the limitation of small sample size and selection bias.

### 2.2 Inclusion and exclusion criteria

**Figure 1:**
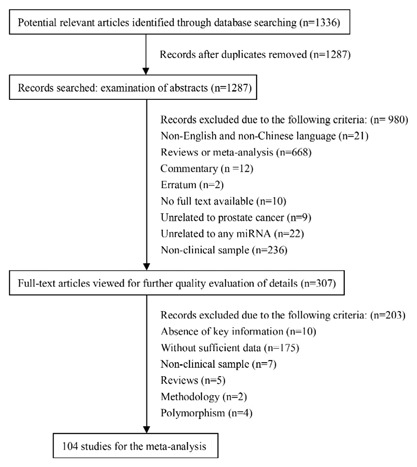
Flowchart of study selection process in this meta‐analysis

More than 1300 articles were retrieved, and 104 publications were included and reviewed in the meta‐analysis (Figure 1). Eligible studies had to fit the following inclusion criteria: (i) a kind of miRNA was involved in the studies; (ii) patients with PC were studied, and gold standard test (eg, histological examination) was used for the PC diagnosis; (iii) prostate tissue or serum or urine samples were used from PC patients or non‐PC patients for miRNA expression comparison; and (iv) validation method and enough patients' information were reported. Eligible studies that met above mentioned criteria were further evaluated and excluded according to a selection process showed in Figure 1. Exclusion criteria were as follows: (i) reviews, letters, commentary, or erratum; (ii) non‐English or non‐Chinese studies; (iii) data was obtained from PC cell lines; (iv) no sufficient data to extract; and (v) duplicate records.

### 2.3 Data extraction

We assessed the data quality of each publication and extracted the following information: (i) basic features, such as first author, publication year, case region, study design, sample number, validation method, and detected miRNAs, as showed in Table 1; (ii) expression levels or fold‐change of detected miRNAs and predictive data, including OS and RFS; and (iii) information needed for quality assessment. If there were no data that could be extracted directly, we used the computer of revman 5.3 software to calculate and generate the relevant data.

**Table 1:** The main characteristics of included studies

First author & publishing year	Region	Study design	Detected samples	Validation	miRNA	Refs.
Robert S. Hudson 2012	USA	PR	A large publicly available data set consisting of 99 primary tumors and 14 distant metastasis and patient data for disease recurrence	qRT‐PCR	miR‐1	32
Yun‐Li Chang 2015	China	P	20 paired of PC tumors and adjacent normal tissues	qRT‐PCR	miR‐7	33
Annika Fendler 2011	Canada Germany	P	52 primary prostate cancers and normal adjacent tissues	qRT‐PCR	miR‐10b	34
Bing Yang 2016	China	P	92 PC 85 BPH 97 controls	qRT‐PCR	miR‐21	16
Christian Melbø‐Jørgensen 2014	Norway	P	535 PC patients 30 patients (14 patients with rapid biochemical failure (BF) and 16 patients without BF) with Gleason score 7	microarray qRT‐PCR ISH	miR‐21	35
Ernest K Amankwah 2013	USA	P	28 recurrent and 37 non‐recurrent prostate cancer cases	qRT‐PCR	miR‐21	28
Judit Ribas 2009	USA	P	10 pairs	Northern blot	miR‐21	36
Marco Folini 2010	Italy	P	36 pairs of PC and N tissues	qRT‐PCR	miR‐21	37
Sabrina Thalita Reis 2012	Brazil	P	53 PC 11 benign prostatic hyperplasia (BPH)	qRT‐PCR	miR‐21	38
Sarvesh Jajoo 2013	USA	P	18 PC	qRT‐PCR	miR‐21	39
Tao Li 2012	China	P	169 radical prostatectomy tissue samples	ISH microarray	miR‐21	40
Wei Huang 2015	China	P	75 localized PC 75 healthy volunteers	qRT‐PCR	miR‐21	17
Yangbo Guan 2016	China	P	85 PC patients and 40 adjacent noncancerous biospy specimens	qRT‐PCR ISH	miR‐21	19
Songwang Cai 2015	China	P	3 pairs of primary human prostate cancer and adjacent non‐tumor tissues 20 pairs of human prostate cancer and adjacent non‐tumor tissues. 123 prostate cancer tissues	Sequencing qRT‐PCR	miR‐23a	41
Hui‐chan He 2012	China	P	4 pairs 20 pairs 26 PC 20N	microarray qRT‐PCR ISH	miR‐23b	42
Shahana Majid 2012	USA	P	118 pairs of laser captured microdisected tissue samples an unmatched group of 27 benign prostatic hyperplasia (BPH) and 20 tumor samples another cohort of 48 samples	qRT‐PCR qMSP ISH	miR‐23b	43
Yusuke Goto 2014	Japan	P	41 noncancerous tissues 49 PC tissues	qRT‐PCR	miR‐23b/27b/24‐1	44
Kai Guo 2016	China	P	140 pairs of fresh PC tissues and normal control tissues	qRT‐PCR	miR‐26a‐5p	45
Xiao‐hui Ling 2014	China	P	103 pairs of prostate tumor tissues and adjacent benign tissues and 28 benign prostate tissues gene expression omnibus (GEO) repository database (http://www.ncbi.nlm.nih.gov/geo/, accession number GSE34932).	qRT‐PCR	miR‐30c	46
Xiao‐Hui Ling 2016	China	P	98 tumor tissue 20 benign prostate hyperplasia (BPH) specimens	qRT‐PCR	miR‐30c	47
Naohito Kobayashi 2012	Japan	P	56 pairs of primary PC and controls	Oligo chips qRT‐PCR	miR‐30d	48
SE Jalava 2012	Finland	P	5 benign prostatic hyperplasia (BPH) and 28 primary PCs 7 BPH and 14 CRPCs	microarray	miR‐32	49
Q. Li 2017	China	P	paired prostate cancer tissue and adjacent normal tissue	qRT‐PCR	miR‐33a	50
Shahana Majid 2013	USA	P	148 matched human tissue samples an unmatched group of 27 benign prostatic hyperplasia (BPH) and 20 tumor samples	qRT‐PCR ISH	miR‐34b	51
Zandra Hagman 2010	Sweden	P	49 PC patients and 25 benign prostatic hyperplasia	qRT‐PCR	miR‐34c	52
Robert S. Hudson 2013	USA	PR	dataset for 28 non‐cancerous tissues, 99 primary tumors and 14 distant metastases with patient data for disease recurrence.	qRT‐PCR	miR‐106b‐25	53
Xu‐Bao Shi 2013	USA	P	19 BPHs, 44 primary CaPs, 6 lymph node metastases, and 10 CR tumors	qRT‐PCR	miR‐124	54
Xiaoke Sun 2013	China	P	A series of 128 cases with PCa	qRT‐PCR	miR‐126	55
Xiaoke Sun 2015	China	P	128 PC tissue and serum and matched controls	qRT‐PCR	miR‐128	56
Song Xu 2015	China	P	98 PC and 56 health controls	qRT‐PCR	miR‐129	57
Song Xu 2016	China	P	118 pairs of PC and noncancerous tissue	qRT‐PCR	miR‐129	58
Xia Li 2014	China	P	135 specimens of patients with prostate cancer, 18 patients with prostatic intraepithelial neoplasia (PIN), and 25 normal prostate tissue samples)	qRT‐PCR ISH	miR‐133b	59
Cheng Pang 2016	China	P	45 PC patients, 45 benign prostatic hyperplasia (BPH) patients and 50 healthy controls serum peripheral whole blood samples	qRT‐PCR	miR‐139‐5p	24
Jason C. Gonzales 2011	USA	P	21 PC	qRT‐PCR	miR‐141	60
Zhuo Li 2015	China	P	20 PCa, 20 BPH, and 20 control volunteers 51 PC and 40 control volunteers	qRT‐PCR	miR‐141	61
M Avgeris 2013	Greece	P	73 radical prostatectomy‐treated PC patients and 64 benign prostate hyperplasia (BPH) patients	qRT‐PCR	miR‐145	62
Bin Xu 2015	China	PR	13 ADPC 9 AIPC MSKCC prostate cancer database (GSE21032)	microarray qRT‐PCR	miR‐146a‐5p	25
Liu Dezhong 2015	China	P	167 PC 4 pairs of PC and adjacent to tumor healthy tissues to tumor	qRT‐PCR	miR‐150	63
Shaniece C. Theodore 2014	USA UK	P	39 pairs of prostate cancer tissues and controls (20 AA and 19 CA) 97 primary tumors and 13 metastases	qRT‐PCR	miR‐152	64
Zsuzsanna Lichner 2013	Canada Germany	P	41 prostatectomy samples were dichotomized to 27 high‐risk and 14 low‐risk The validation set: 35 high‐risk patients and 29 low‐risk patients	microarray qRT‐PCR	miR‐152	65
Ranlu Liu 2013	China	P	5 PC 3 BPH	array	miR‐182	66
Katsuki Tsuchiyama 2013	Japan	P	patient set 1: 22 GP 3, 35 GP 4, and 12 GP 5 patient set 2: 10 GP 4 Cancer tissues from each GP and adjacent normal counterparts were separately collected using LCM	qRT‐PCR	miR‐182‐5p	67
Hongtuan Zhang 2015	China	P	180 pairs of PC and adjacent noncancerous tissues	qRT‐PCR	miR‐188‐5p	68
Amelie Hailer 2014	Germany	P	15 BPH 161 PC 17 LNM	qRT‐PCR	miR‐203	69
Berlinda Verdoodt 2013	Germany	P	111 pairs of formalin‐fixed paraffin‐embedded (FFPE) prostatectomy specimens with primary prostate adenocarcinoma (PCa) and control	qRT‐PCR	miR‐205	70
Charis Kalogirou 2013	Germany Belgium	P	105 HRPCa for study collective and 10 BHP 78 HRPCa for validation	qRT‐PCR	miR‐205	71
Sigve Andersen 2016	Norway	P	535 prostatectomy patients	microarray ISH	miR‐210	72
Aida Gordanpour 2011	Canada	P	153 radical prostatectomy samples	microarray qRT‐PCR	miR‐221	73
Burkhard Kneitz 2014	Germany Belgium	P	cohort 1, *N* = 134; cohort 2, *n* = 89	qRT‐PCR	miR‐221	74
Yongbao Wei 2014	China	P	10 pairs of PC tissues and adjacent non‐cancerous tissues	qRT‐PCR	miR‐223‐3p	75
Hao Fu 2015	China	PR	A 4 and 20 pairs of primary PC and adjacent non‐tumor frozen samples the Taylor dataset (149 primary PC tissues and 29 adjacent non‐cancerous prostate tissues)	array qRT‐PCR	miR‐224	76
Konstantinos Mavridis 2013	Greece	P	66 BPH or 73 CaP	qRT‐PCR	miR‐224	77
Zhuo‐Yuan Lin 2014	China USA	P	4 and 20 pairs of primary PC and adjacent non‐tumor frozen samples Human PC tissue microarrays (TMA) consisting 114 PC tissues respectively from Caucasian and African‐American PC patients	array qRT‐PCR ISH	miR‐224	78
Jian‐Jun Wei 2011	USA	P	TMA100 contained 100 PC cases (from the Cooperative PC Tissue Resource at New York University) and TMA96 contained 96 cases (from Northwestern University).	microarray qRT‐PCR ISH	miR‐296	79
Chendil Damodaran 2016	USA	P	58 FFPE 4 metastatic tumors, 6 fresh tumor tissues and 13 BPH	qRT‐PCR	miR‐301a	80
Robert K. Nam 2016	Canada	P	585 prostate cancer	qRT‐PCR	miR‐301a	81
Si‐wei Xiong 2013	China	P	20 clinical PC tissues 104 clinical PC tissues	qRT‐PCR ISH	miR‐335	82
Sven Wach 2015	Germany	P	146 PC patients, 35 benign prostate hyperplasia (BPH) patients and 18 healthy controls serum	qRT‐PCR	miR‐375	31
Yuan Wang 2016	USA	R	495 tumor tissues and 52 normal tissues from TCGA data	qRT‐PCR	miR‐375	83
N Bucay 2017	USA	PR	TCGA(187 primary prostate adenocarcinoma cases) validation cohort: 112 PC FFPE tissues and matched adjacent normals	qRT‐PCR	miR‐383	84
Martin Mørck Mortensen 2014	Denmark	P	36 prostate cancer Samples 163 radical prostatectomy patients 40 patients (20 recurrent and 20 non‐recurrent patients)	qRT‐PCR	miR‐449b	85
Melissa Colden 2017	USA	PR	48 pairs of LCM tissue samples validation cohort: 56 prostate adenocarcinoma (TCGA database)	qRT‐PCR	miR‐466	86
X. M. Tian 2017	China	P	20 prostate cancer tumor tissues 20 tumor‐adjacent tissues and 20 normal prostate tissues	qRT‐PCR	miR‐509‐5p	87
Jayant K. Rane 2015	UK	P	5 benign prostatic hyperplasia 5 G7 prostate cancer, and 3 castration‐resistant PC (CRPC)	microarray	miR‐548c‐3p	88
Takeshi Chiyomaru 2013	USA	P	48 pairs of PC tissues and adjacent non‐cancerous tissues	microarray qRT‐PCR	miR‐574‐3p	89
Ze‐Hua Zuo 2015	USA	P	77 organ donor (OD) prostates, 324 benign prostate tissues adjacent to cancer, and 216 PCs	qRT‐PCR ISH	miR‐650	90
Li Jiao 2014	China	P	127 patients with prostate cancer and 10 patients with benign prostatic hyperplasia (BPH)	qRT‐PCR microarray ISH	miR‐663	91
Sharanjot Saini 2012	USA	P	40 PC and 8 normal 96 paired	ISH qRT‐PCR	miR‐708	92
Dibash K. Das 2016	USA	P	404 PC (389 CA and 15 AA)	qRT‐PCR	miR‐1207‐3p	93
Nathan Bucay 2016	USA	P	100 pairs of PC and adjacent normals	qRT‐PCR	miR‐3622b	94
Yang Wang 2016	China	P	3 CRPC and 3 ADPC samples 30 ADPC tissues and 18 CRPC tissues	microarray qRT‐PCR	miR‐4638‐5p	95
Albertoivan S. Guadarrama 2016	Mexico	P	73 PC urine and 70 BPH urine	qRT‐PCR	miR‐100 miR‐200b	96
Betina Katz 2014	Brazil	P	51 localized prostate cancer (PCa)	qRT‐PCR	miR‐30a miR‐200b	15
Chunjiao Song 2015	China	P	7 G>7 8 G7 9 Non‐cancerous Samples 7 8 9 12 G>7 12 G7 12 BPH	sequencing qRT‐PCR	miR‐125b‐5p miR‐126‐5p miR‐151a‐5p miR‐221‐3p miR‐222‐3p miR‐486‐5p	97
Darina Kachakova 2015	Bulgaria	P	59 prostate cancer (PC) patients and two groups of controls: 16 benign prostatic hyperplasia (BPH) samples and 11 young asymptomatic men	qRT‐PCR	miR‐30c miR‐141 miR‐375 let‐7c	98
D Lin 2011	China	P	35 PC (17 aggressive and 18 non‐aggressive)	qRT‐PCR	miR‐221 miR‐222	99
Fulya Yaman Agaoglu 2011	Turkey	P	51 PC (26 local/local advanced or 25 metastatic PCa) 20 healthy individuals	qRT‐PCR	miR‐21 miR‐141 miR‐221	100
Heather H. Cheng 2013	USA	P	25 mCRPC and 25 healthy donor serum pools the sera of an additional 21 mCRPC patients and 20 age‐matched healthy Controls for validation	array qRT‐PCR	miR‐141 miR‐200a/c miR‐210 miR‐375	20
Hui‐Ming Lin 2017	Australia	P	Phase 1 cohort: 97 patients Phase 2 cohort: 89 patients	qRT‐PCR	miR‐20a/20b miR‐21 miR‐25 miR‐132 miR‐145a miR‐200a/b/c miR‐222 miR‐301b miR‐375 miR‐429d miR‐590‐5p	29
Irene Casanova‐Salas 2014	Spain	PR	10 normal prostate and 50 prostate cancer samples an independent cohort of 273 paraffin embedded prostate cancer samples Another 92 urine samples GEO (Gene Expression Omnibus) database Accession No. GSE45604 (http://www.ncbi.nlm.nih.gov/geo/)	qRT‐PCR	miR‐182 miR‐187	101
Ivan D. Osipov 2016	Russia	P	Blood samples from 47 healthy donors and 48 prostate cancer (PC) patients	qRT‐PCR	miR‐141 miR‐205	102
Jorge Torres‐Ferreira 2017	Portugal	P	180 localized PC and 15 control 95 urine sediments and 46 controls 74 prostate biopsies	Human Methylation 450 Bead Chip qMSP	miR‐34b/c miR‐129‐2 miR‐152 miR‐193b miR‐663a miR‐1258	103
Katia R. M. Leite 2011‐1	Brazil	P	18 localized high grade prostate carcinoma (PC) with mean Gleason score 8.6, all staged pT3 4 patients with metastatic, androgen‐independent prostate carcinoma 6 nonneoplastic tissue (benign prostate hyperplasia)	qRT‐PCR	miR‐100 miR‐218 Let‐7c	26
Katia R. M. Leite 2011‐3	Brazil	P	49 prostate cancer (28 men without and 21 with biochemical recurrence)	qRT‐PCR	miR‐100 miR‐145 miR‐191	27
Katia R. M. Leite 2013	Brazil	P	63 localized prostate carcinoma 15 high grade prostate intraepithelial neoplasia (HGPIN) 14 localized favorable CaP and 34 unfavorable, mostly non‐organ‐confined disease.	qRT‐PCR	miR‐21 miR‐206	22
Kristina Stuopelytė 2016	Lithuania	P	13 PC 143 urine PC and 23 urine BPH 52 PC and 12 N	microarray qRT‐PCR	miR‐19a/b miR‐21 miR‐95	104
Kristina Stuopelyte 2016	Lithuania	P	56 Cancerous and 16 non‐cancerous 215 PC 23 benign prostatic hyperplasia and 62 asymptomatic controls	array qRT‐PCR	miR‐148a miR‐375	105
Maria Giulia Egidi 2015	Italy	P	35 urine sediments of PC and 26 benign prostatic hyperplasia (BPH).	qRT‐PCR	miR‐25 miR‐191 miR‐200b miR‐452	106
Maria Schubert 2013	Germany Belgium	P	cohort A: 98 high‐risk PC Cohort B: 92 FFPE samples from RP Cohort C: 21 pairs of PC tissues and adjacent benign tissues	microarray qRT‐PCR	miR‐146b miR‐181b miR‐361 miR‐515‐3p/5p let‐7a/b/c	107
Matthew J. Roberts 2015	Australia Germany		20 specimens 54 non‐cancerous histology and 98 cancer tissues	qRT‐PCR	miR‐125b miR‐200b/c miR‐375	108
Robert Mahn 2011	Germany	P	37 localized PC 18 BPH 8 metastatic PC 20 healthy volunteers 10 PC and adjacent tissues and pre/post prostatectomy serum	qRT‐PCR	miR‐16 miR‐26a miR‐32 miR‐195 Let‐7i	109
Stefan Ambs 2008	USA	P	60 primary prostate tumors and 16 non‐tumor prostate tissues	qRT‐PCR microarray	miR‐1 miR‐32 miR‐106a/106b	110
Taha A Haj‐Ahmad 2014	Egypt	PR	8 PC patients, 12 BPH patients and 10 healthy males urine samples	microarray qRT‐PCR	miR‐484 miR‐1825	111
Tong Sun 2012	USA	P	86 individuals, prostate tumor tissues from 34 individuals with localized hormone naïve disease, and bone‐derived metastatic CRPC tissues from 17 individuals.	qRT‐PCR	miR‐23b/27b miR‐221/222	112
William T. Budd 2015	USA	P	4 pairs of frozen PC and BPH tissue samples 1 FFPE prostate sample	qRT‐PCR	miR‐22 miR‐125b	113
Xiaoyi Huang 2015	USA	P	23 CRPC patients 100 CRPC	sequencing qRT‐PCR	miR‐375 miR‐1290	30
Yubin Hao 2011	China	P	20 human prostate specimens (8 prostate cancer tissues and 12 benign prostatic hyperplasia tissues	qRT‐PCR	miR‐16 miR‐21 miR‐34c miR‐101 miR‐125b miR‐141	18
Beatriz A. Walter 2013	USA	P	37 matched prostate tumors, normal epithelium and adjacent stroma. 40 PC 10 N 10stroma	microarray qRT‐PCR	34 deregulated	23
Fan Feng 2017	Spain	R	Dataset (GSE45604) 50 PC and 10 normal specimens urine	data analysis	7 up 59 down	21
Rihan El Bezawy 2017	Italy	P	44 pairs of PC specimens and normal tissues (GSE76260)	qRT‐PCR	5 up 13 down	114
Robert K. Nam 2015	Canada.	P	546 prostate cancer	qRT‐PCR	29 up 4 down	115
Yanan Sun 2016	Nonchina	R	3 microarray studies: 197 samples of PC and 43 samples of normal control	data analysis	10 up 19 down	116

P, prospective study; R, retrospective study; ISH, in situ hybridization; Refs, references; PC, prostate cancer; BPH, benign prostate hyperplasia; NM, not mentioned; LCM, laser‐captured microdissection; CRPC, castration resistant prostate cancer.

### 2.4 Statistical analysis

We drew forest plots to estimate miRNAs expression levels in PC and control patients' samples, and their effects on PC patients' OS and RFS. Publication bias was explored by funnel plots.^12^, ^13^ The fixed‐effects model was used to calculate HR and 95% CI in all enrolled studies.^14^ We used Chisquared and the inconsistency index (*I* ^2^) tests to assess the heterogeneities (*P* value ≤0.1 and *I* ^2^ value ≥50 %). To avoid the influence of heterogeneity, subgroup analyses were performed based on the characteristics of included studies, such as patients' ethnicities, pathological types, and detected sample types, etc. All *P* values were two‐tailed and a *P* value <0.05 was considered to be statistically significant.

## 3 RESULTS

### 3.1 Summary of included studies

**Figure 2:**
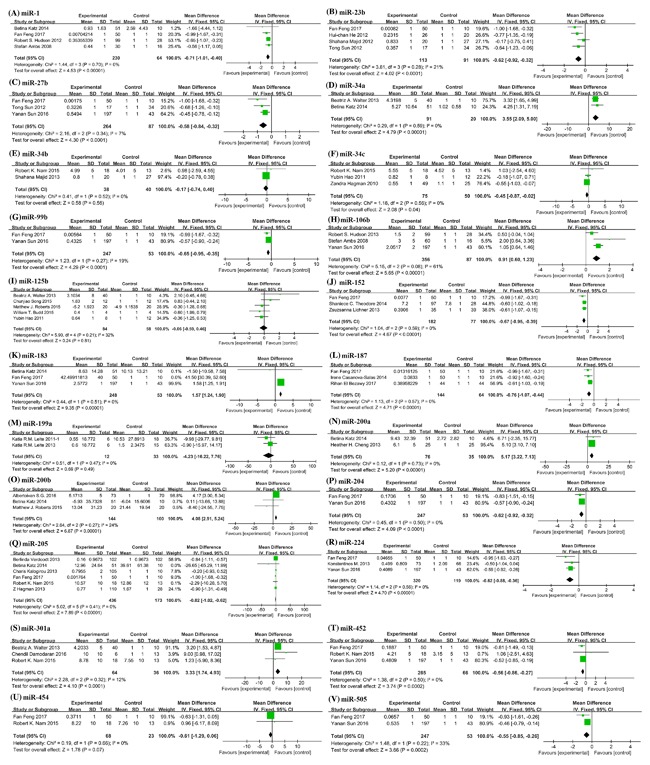
Forest plots showing mean expression levels of different miRNAs with corresponding heterogeneity statistics. (A) miR‐1; (B) miR‐23b; (C) miR‐27b; (D) miR‐34a; (E) miR‐34b; (F) miR‐34c; (G) miR‐99b; (H) miR‐106b; (I) miR‐125b; (J) miR‐152; (K) miR‐183; (L) miR‐187; (M) miR‐199a; (N) miR‐200a; (O) miR‐200b; (P) miR‐204; (Q) miR‐205; (R) miR‐224; (S) miR‐301a; (T) miR‐452; (U) miR‐454; (V) miR‐505. Squares and horizontal lines correspond to study‐specific HRs and 95% CIs; respectively. The area of the squares correlates the weight of each enrolled study and the diamonds represent the summary HRs and 95% CIs

**Figure 3:**
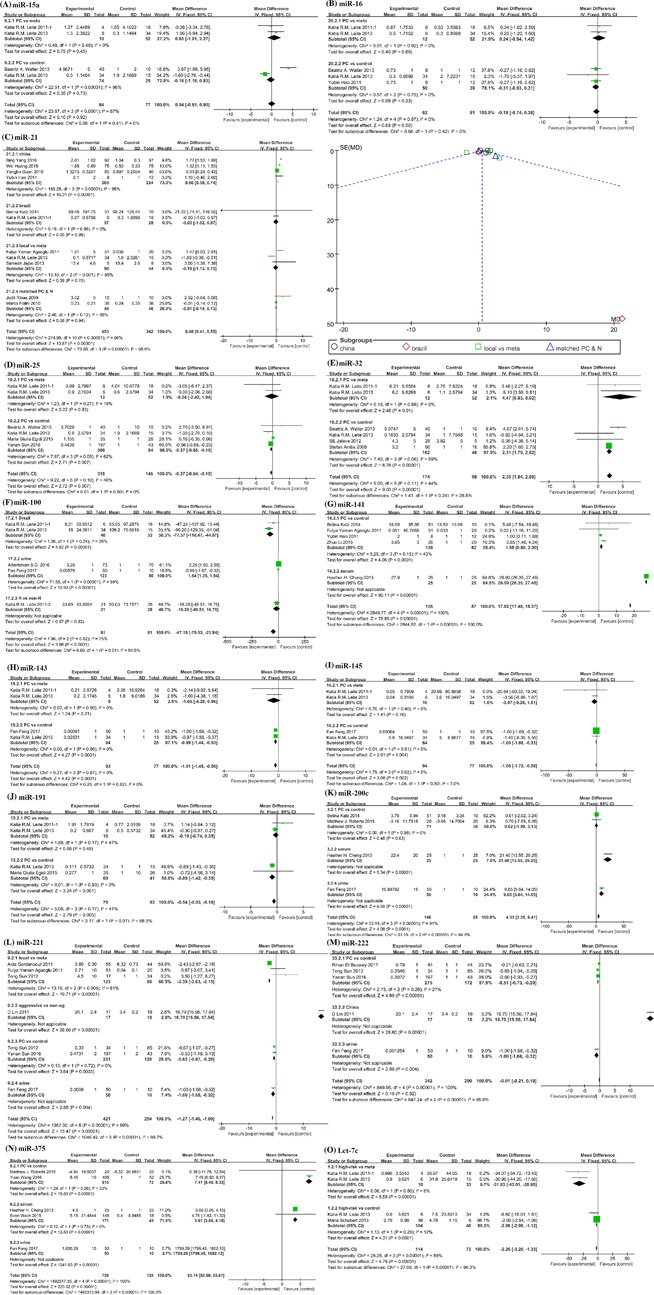
Forest plots of subgroup analyses stratified by ethnicities; main pathologic types and detected samples; showing mean expression levels or fold change with corresponding heterogeneity statistics. (A) miR‐15a; (B) miR‐16; (C) forest plot and funnel plot of miR‐21; each point represents a separate study for publication bias test in funnel plot; (D) miR‐25; (E) miR‐32; (F) miR‐100; (G) miR‐141; (H) miR‐143; (I) miR‐145; (J) miR‐191; (K) miR‐200c; (L) miR‐221; (M) miR‐222; (N) miR‐375; (O) let‐7c. Squares and horizontal lines correspond to study‐specific HRs and 95% CIs; respectively. The area of the squares correlates the weight of each enrolled study and the diamonds represent the summary HRs and 95% CIs

**Figure 4:**
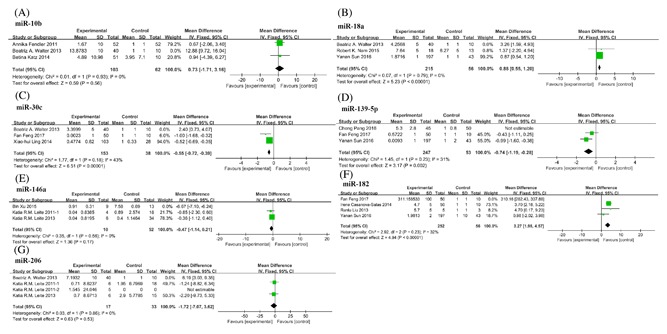
Forest plots showing mean expression levels of miRNAs with corresponding heterogeneity statistics. (A) miR‐10b; (B) miR‐18a; (C) miR‐30c; (D) miR‐139‐5p; (E) miR‐146a; (F) miR‐182; (G) miR‐206. Squares and horizontal lines correspond to study‐specific HRs and 95% CIs; respectively. The area of the squares correlates the weight of each enrolled study and the diamonds represent the summary HRs and 95% CIs

**Figure 5:**
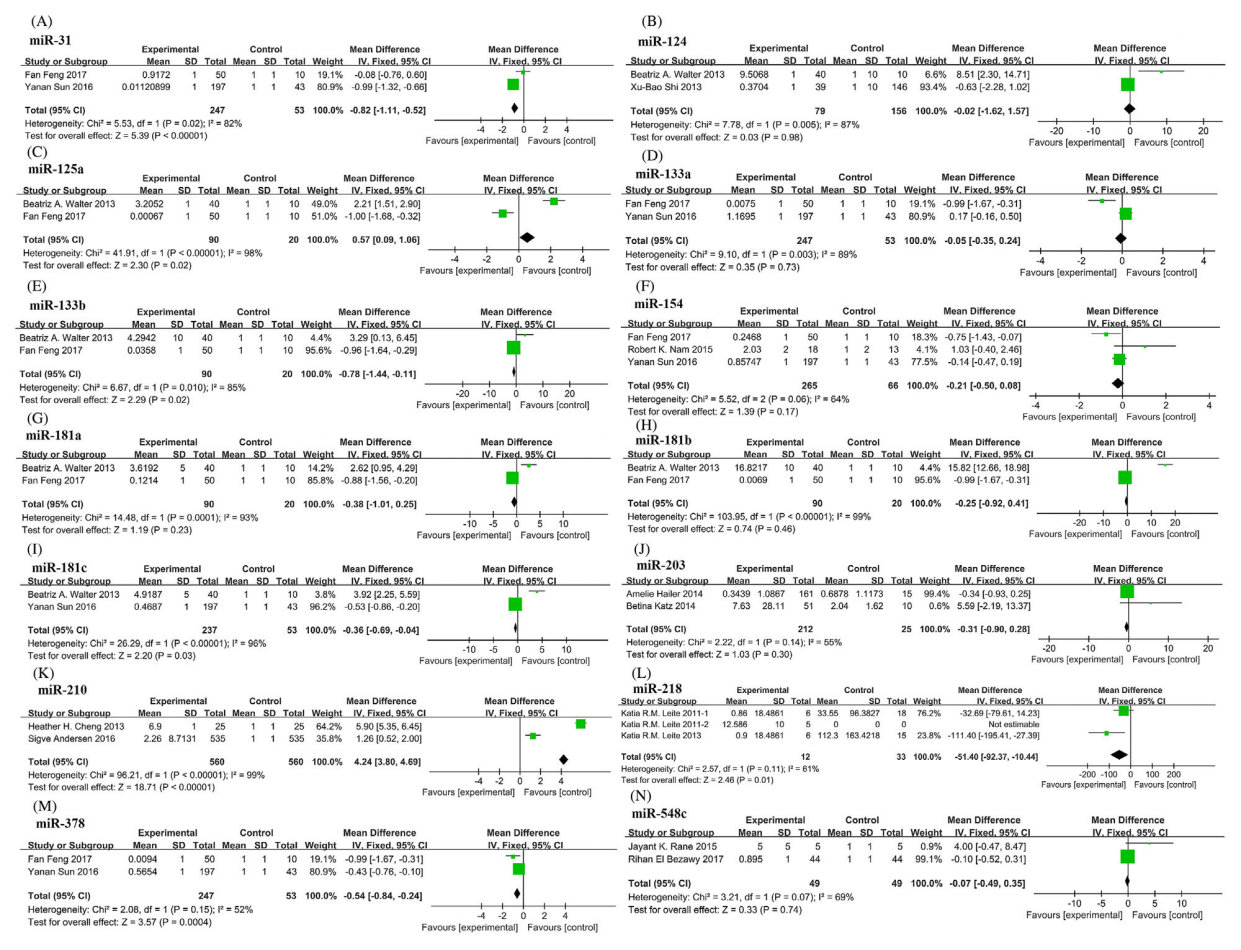
Forest plots showing mean expression levels of miRNAs with significant heterogeneity. (A) miR‐31; (B) miR‐124; (C) miR‐125a; (D) miR‐133a; (E) miR‐133b; (F) miR‐154; (G) miR‐181a; (H) miR‐181b; (I) miR‐181c; (J) miR‐203; (K) miR‐210; (L) miR‐218; (M) miR‐378; (N) miR‐548c. Squares and horizontal lines correspond to study‐specific HRs and 95% CIs; respectively. The area of the squares correlates the weight of each enrolled study and the diamonds represent the summary HRs and 95% CIs

**Figure 6:**
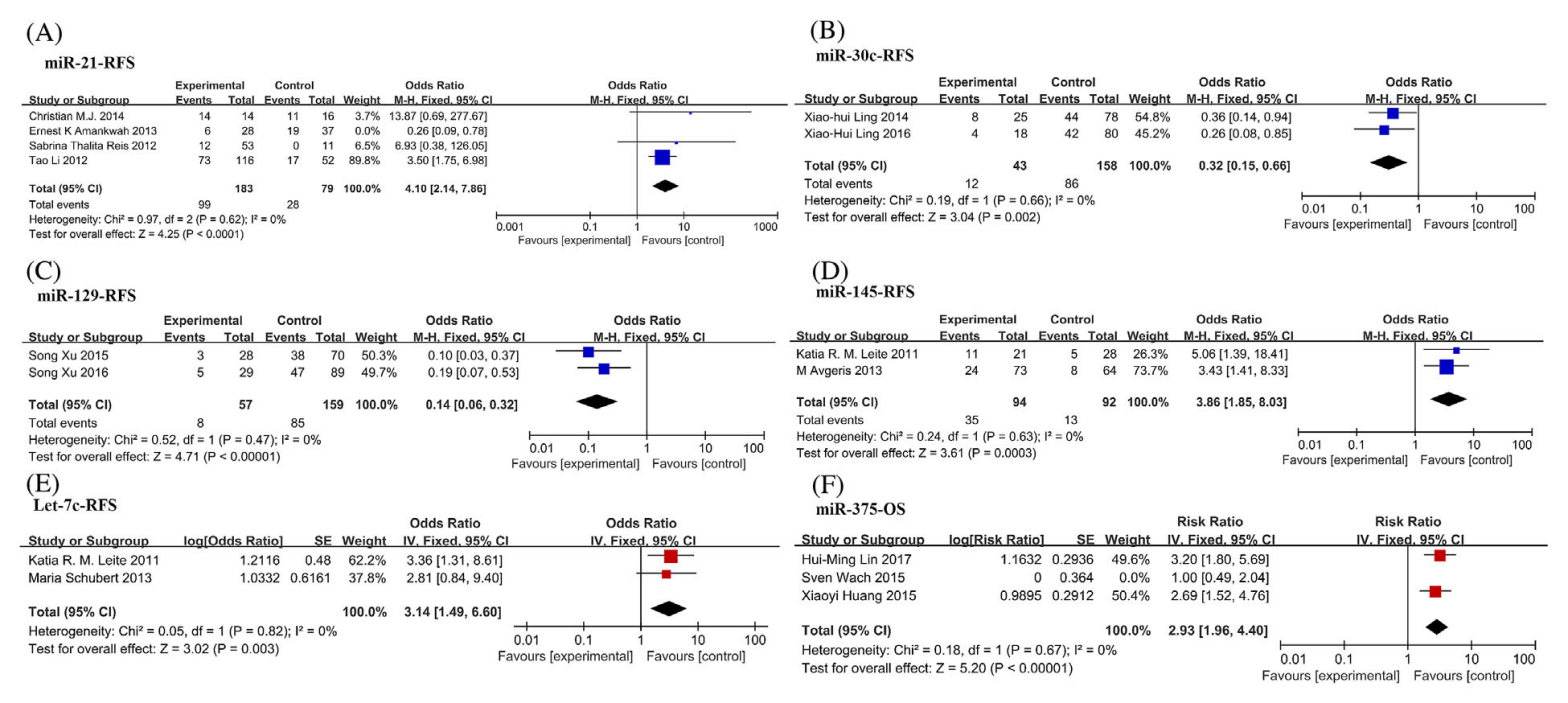
Forest plots for merged analyses of recurrence‐free survival (RFS) and overall survival (OS) associated with different miRNAs expression. Forest plots for RFS analyses of (A) miR‐21 (B) miR‐30c; (C) miR‐129; (D) miR‐145; (E) let‐7c; (F) Forest plots of OS analyses of miR‐375. Squares and horizontal lines correspond to study‐specific HRs and 95% CIs; respectively. The area of the squares correlates the weight of each enrolled study and the diamonds represent the summary HRs and 95% CIs

A total of 1336 primary literatures were searched in PubMed, Embase, Cochrane, and CNKI. As shown in the selection process (Figure 1), we firstly removed 49 studies due to duplication. Then, we excluded 980 and 202 studies, respectively, after abstracts and full texts were reviewed. Ultimately, only 104 articles were considered eligible for the meta‐analysis. The characteristics of 104 included studies were summarized in Table 1 in alphabetical order of the miRNAs. The publication years of these records ranged from 2007 to 2017. In these 104 studies, some were divided into several parts because of multiple miRNAs. Data of enrolled records were collected from the United States, China, Germany, Greece, Italy, Austria, Korea, and Brazil, etc. The dominant ethnicity was Caucasian in more than half of studies, while 38‐2 studies were executed in Asians. Most studies were prospective in design. The expression level of miRNA was usually detected by quantitative real‐time polymerase chain reaction (qRT‐PCR) and microarray in tissue samples, while 6 + 2 studies were in serum or plasma samples, 6 + 2 studies were in urine (Table 1). Among these studies, 71 records were associated with Mean ± SD and fold‐change of miRNA expression level in tumor or control samples (Table 2 and Figures 2, 3, 4, 5). A 29 focused on RFS (Table 3 and Figure 6A‐E), and 11 focused on OS (Table 4 and Figure 6F). In the analysis of RFS and OS, 26, and 9 records directly reported HRs and 95% CIs, respectively, while in other studies we extrapolated these necessary variables by available original data (Tables 3, 4 and Figure 6).

**Table 2:** The expression levels of miRNAs

miRNA	Samples	Mean ± SD (PC vs control)	Fold change (PC/control)	*P* value	Refs
miR‐7	20 pairs of tumors and adjacent normal tissues	1.7 ± 1.04 vs 1.21 ± 0.55		0.6569	33
miR‐7‐2*	44 pairs of PC and normal		0.806642	2.19E‐02	114
miR‐7c	50 PC and 10 normal		0.001272	1.56E‐02	21
miR‐9	51 localized PC	0.96 ± 0.89 vs 1.34 ± 2.47		0.637	15
miR‐9‐1	18 PC with recurrence and 13 PC no metastasis no recurrence	5.9 vs 4.98		0.04723	115
miR‐9‐2	18 PC with recurrence and 13 PC no metastasis no recurrence	5.89 vs 4.97		0.04892	115
miR‐9‐3	18 PC with recurrence and 13 PC no metastasis no recurrence	6.12 vs 4.96		0.01907	115
miR‐15b	40 PC and 10 normal		3.4761	0.0418	23
miR‐18b	40 PC and 10 normal		6.8061	0.0133	23
miR‐20b	40 PC and 10 normal		3.1928	0.0501	23
miR‐22	4 frozen tissue samples 1 FFPE prostate sample		3.2	NM	113
miR‐24	50 PC and 10 normal		0.27	3.68E‐03	21
miR‐24‐2	50 PC and 10 normal		0.164459	9.76E‐03	21
miR‐26a‐5p	140 pairs of fresh PC tissues and normal tissues	0.058 ± 0.016 vs 0.115 ± 0.043		<0.001	45
miR‐28‐3p	50 PC and 10 normal		0.00668	1.08E‐02	21
miR‐28‐5p	50 PC and 10 normal		0.003839	3.28E‐03	21
miR‐29b	51 localized PC	0.51 ± 0.64 vs 0.56 ± 0.77		0.852	15
miR‐30a	51 localized PC	6.37 ± 7.91 vs 1.7 ± 2.77		0.039	15
miR‐30c‐1	50 PC and 10 normal		0.257951	3.18E‐02	21
miR‐30d	56 pairs of primary PC and control	7.95 ± 7.03 vs 6.23 ± 6.06		0.03	48
miR‐30e*	44 pairs of PC and normal		0.840896	4.10E‐03	114
miR‐33a	Paired prostate cancer tissue and adjacent normal tissue		0.1389	<0.01	50
miR‐34c‐3p	50 PC and 10 normal		0.17691	7.42E‐03	21
miR‐34c‐5p	40 PC and 10 normal		8.0395	0.0283	23
miR‐92a	40 PC and 10 normal		3.0015	0.0177	23
miR‐93	197 PC and 43 normal		2.14	1.69E‐09	116
miR‐96	197 PC and 43 normal		2.35	2.33E‐12	116
miR‐101	8 PC and 12 BPH		0.91	>0.05	18
miR‐122	40 PC and 10 normal		5.5663	0.0054	23
miR‐126	128 PCa	1.05 ± 0.63 vs 2.92 ± 0.98		<0.001	55
miR‐126‐5p	12 G > 7, 12 G7, and 12 non‐cancerous samples		2.22	<0.05	97
miR‐128	128 PC tissue and serum and matched normal	1.05 ± 0.63 vs 2.92 ± 0.98		<0.001	56
miR‐128a	40 PC and 10 normal		4.5004	0.0143	23
miR‐130b	197 PC and 43 normal		1.974463	3.52E‐07	116
miR‐134	40 PC and 10 normal		23.1323	0.0125	23
miR‐135b	40 PC and 10 normal		4.0019	0.0141	23
miR‐138‐2	18 PC with recurrence and 13 PC no metastasis no recurrence	5.23 vs 4.25		0.03941	115
miR‐139	18 PC with recurrence and 13 PC no metastasis no recurrence	7.24 vs 8.08		0.03061	115
miR‐146b‐5p	40 PC and 10 normal		3.5577	0.0019	23
miR‐148b	40 PC and 10 normal		2.8135	0.0358	23
miR‐149	44 pairs of PC and normal		0.796	0.416	114
miR‐151a‐5p	12 G > 7, 12 G7, and 12 non‐cancerous samples		2.02	<0.05	97
miR‐153	197 PC and 43 normal		3.1425	2.74E‐13	116
miR‐155	51 localized PC	3.12 ± 4.56 vs 2.09 ± 3.8		0.463	15
miR‐181d	50 PC and 10 normal		0.062341	9.34E‐03	21
miR‐182‐5p	patient set 1:69 PC patient set 2:10 PC	Patient set 1: 1.745 ± 0.278 vs 0.864 ± 0.136 Patient set 2: 1.863 ± 0.381 vs 0.761 ± 0.158		0.021	66
miR‐183*	44 pairs of PC and normal		1.505247	7.68E‐03	114
miR‐184	40 PC and 10 normal		4.0633	0.0086	23
miR‐188	18 PC with recurrence and 13 PC no metastasis no recurrence	8.48 vs 7.5		0.01878	115
miR‐188‐5p	180 pairs of PC and normal		0.0956	NM	68
miR‐193a‐5p	40 PC and 10 normal		4.5984	0.0094	23
miR‐193b	40 PC and 10 normal		12.649	0.0021	23
miR‐199a‐1	50 PC and 10 normal		0.451942	2.06E‐02	21
miR‐199a‐3p	50 PC and 10 normal		0.000759	1.08E‐02	21
miR‐214	40 PC and 10 normal		9.9075	0.0055	23
miR‐215	40 PC and 10 normal		8.4863	0.038	23
miR‐220a	44 pairs of PC and normal		0.907519	0.355	114
miR‐221‐3p	12 G > 7, 12 G7, and 12 non‐cancerous samples		5.47	<0.05	97
miR‐222‐3p	12 G > 7, 12 G7, and 12 non‐cancerous samples		3.88	<0.05	97
miR‐223	18 PC with recurrence and 13 PC no metastasis no recurrence	10.66 vs 11.9		0.00179	115
miR‐223‐3p	10 pairs of PC and adjacent non‐cancerous tissues	2.98 ± 1.45 vs 1.55 ± 0.38		<0.01	75
miR‐296	TMA100: 100 PC cases TMA96: 96 cases	1.79 ± 0.19 vs 2.71 ± 0.16		<0.05	79
miR‐296‐5p	44 pairs of PC and normal		0.646176	1.49E‐02	114
miR‐301b	18 PC with recurrence and 13 PC no metastasis no recurrence	4.61 vs 3.65		0.02116	115
miR‐320c‐2	18 PC with recurrence and 13 PC no metastasis no recurrence	3.54 vs 2.39		0.0393	115
miR‐324‐5p	197 PC and 43 normal		0.565156	2.06E‐05	116
miR‐328	197 PC and 43 normal		0.511	7.85E‐07	116
miR‐335	20 pairs of primary PC and adjacent 104 PC and 20 benign	3.27 ± 0.99 vs. 4.55 ± 1.34		<0.05	82
miR‐338‐5p	50 PC and 10 normal		14.70974	9.88E‐03	21
miR‐362‐3p	50 PC and 10 normal		0.265027	3.18E‐02	21
miR‐372	40 PC and 10 normal		6.8639	0.0184	23
miR‐373	51 localized PC	0.26 ± 0.37 vs. 0.29 ± 0.32		0.186	15
miR‐376a	50 PC and 10 normal		0.457502	1.41E‐02	21
miR‐378*	197 PC and 43 normal		0.476022	1.64E‐08	116
miR‐378c	50 PC and 10 normal		0.011878	1.40E‐03	21
miR‐381	50 PC and 10 normal		0.20897	2.30E‐02	21
miR‐383	TCGA:187 primary PC validation cohort: 112 pairs of PC and adjacent normals		0.25	0.05	84
miR‐411	18 PC with recurrence and 13 PC no metastasis no recurrence	3.83 vs 2.73		0.02673	115
miR‐421	50 PC and 10 normal		0.03487	6.02E‐04	21
miR‐422a	50 PC and 10 normal		0.014149	8.65E‐05	21
miR‐424	50 PC and 10 normal		0.088399	2.94E‐02	21
miR‐429	51 localized PC	7.74 ± 7.34 vs 7.75 ± 17.18		0.998	15
miR‐433	18 PC with recurrence and 13 PC no metastasis no recurrence	4.21 vs 3.15		0.030601	115
miR‐455‐3p	50 PC and 10 normal		0.001986	2.07E‐02	21
miR‐455‐5p	50 PC and 10 normal		0.093956	9.76E‐03	21
miR‐485‐3p	50 PC and 10 normal		0.2564	1.82E‐02	21
miR‐486	18 PC with recurrence and 13 PC no metastasis no recurrence	4.49 vs 5.6		0.03746	115
miR‐486‐5p	12 G > 7, 12 G7, and 12 non‐cancerous samples		0.3937	<0.05	97
miR‐487b	197 PC and 43 normal		0.565379	3.69E‐05	116
miR‐489	18 PC with recurrence and 13 PC no metastasis no recurrence	3.66 vs 2.67		0.02183	115
miR‐490‐5p	50 PC and 10 normal		0.184615	1.56E‐02	21
miR‐495	51 localized PC	0.77 ± 0.39 vs 0.93 ± 0.32		0.78	15
miR‐497	18 PC with recurrence and 13 PC no metastasis no recurrence	11.19 vs 10.28		0.01111	115
miR‐501	18 PC with recurrence and 13 PC no metastasis no recurrence	5.74 vs 4.91		0.00525	115
miR‐502‐5p	197 PC and 43 normal		0.573804	3.86E‐05	116
miR‐503	50 PC and 10 normal		0.376508	1.41E‐02	21
miR‐507	PC and matched Normal		0.858565	4.88E‐03	114
miR‐509‐3‐5p	50 PC and 10 normal		0.318223	3.62E‐02	21
miR‐509‐5p	20 PC, 20 tumor‐adjacent tissues, and 20 normal prostate tissues	0.314 ± 0.048 vs 1.532 ± 0.015		<0.05	87
miR‐518b	44 pairs of PC and normal		0.779165	4.23E‐02	114
miR‐543	50 PC and 10 normal		0.270522	1.08E‐02	21
miR‐545	18 PC with recurrence and 13 PC no metastasis no recurrence	5.1 vs 4.04		0.00524	115
miR‐574‐3p	48 pairs of PC and adjacent non‐cancerous tissues		0.5	<0.0001	89
miR‐612	44 pairs of PC and normal		1.658639	5.31E‐03	114
miR‐624	18 PC with recurrence and 13 PC no metastasis no recurrence	5.66 vs 4.07		0.030601	115
miR‐628‐3p	50 PC and 10 normal		0.04014	1.80E‐03	21
miR‐650	216 PC, 324 benign, and 77 control 22 PC, 20 benign, and 11 control	1.29 ± 0.08 vs 1.07 ± 0.05		0.012	90
miR‐652	18 PC with recurrence and 13 PC no metastasis no recurrence	8.3 vs 6.73		0.00124	115
miR‐659	44 pairs of PC and normal		0.795536	4.10E‐03	114
miR‐663	197 PC and 43 normal		0.545382	2.62E‐09	116
miR‐671	18 PC with recurrence and 13 PC no metastasis no recurrence	7.75 vs 6.94		0.00072	115
miR‐708	18 PC with recurrence and 13 PC no metastasis no recurrence	6.67 vs 5.45		0.01206	115
miR‐875‐3p	50 PC and 10 normal		0.383402	3.12E‐02	21
miR‐875‐5p	44 pairs of PC and normal		0.632878	1.31E‐02	114
miR‐887	50 PC and 10 normal		0.211747	2.33E‐02	21
miR‐1184	50 PC and 10 normal		3.450542	1.56E‐02	21
miR‐1206	44 pairs of PC and normal		0.907519	2.68E‐02	114
miR‐1207‐3p	PC patients of 389 CA and 15 AA	Black: 3.00 ± 2.65 White 5.36 ± 3.76		0.062	93
miR‐1207‐5p	50 PC and 10 normal		180.2841	4.72E‐02	21
miR‐1228	44 pairs of PC and normal		1.086735	4.90E‐02	114
miR‐1238	50 PC and 10 normal		0.883057	2.36E‐02	21
miR‐1244	44 pairs of PC and normal		1.484524	5.20E‐03	114
miR‐1245	44 pairs of PC and normal		1.265757	3.01E‐02	114
miR‐1248	18 PC with recurrence and 13 PC no metastasis no recurrence	8.91 vs 7.77		0.01907	115
miR‐1249	18 PC with recurrence and 13 PC no metastasis no recurrence	5.37 vs 4.47		0.00622	115
miR‐1271	50 PC and 10 normal		0.02573	3.28E‐03	21
miR‐1302‐1	18 PC with recurrence and 13 PC no metastasis no recurrence	4.53 vs 2.75		0.01529	115
miR‐1302‐3	18 PC with recurrence and 13 PC no metastasis no recurrence	4.42 vs 2.75		0.01529	115
miR‐1302‐7	18 PC with recurrence and 13 PC no metastasis no recurrence	4.19 vs 2.55		0.02113	115
miR‐3200‐3p	50 PC and 10 normal		0.40029	4.48E‐02	21
miR‐4288	50 PC and 10 normal		0.219167	3.76E‐03	21
miR‐4328	50 PC and 10 normal		0.470068	4.96E‐02	21
miR‐4638‐5p	3 CRPC and 3 ADPC 18 CRPC and 30 ADPC		0.4167 0.2128	1.44E‐08	95
let‐7b	Cohort A: 6 BPH tissues and 13 high‐risk PC specimens Cohort B: 92 FFPE PC samples Cohort C: 21 pairs of fresh frozen PC tissue and adjacent benign tissue	3.16 ± 0.76 vs 3.8 ± 0.37		<0.01	107

Refs, reference; PC, prostate cancer; BPH, benign prostate hyperplasia; CRPC, castration resistant prostate cancer.

**Table 3:** The recurrence‐free survival of miRNAs in enrolled studies

miRNA	Samples	RFS HR/RR (95%CI)	*P* value	Refs
miR‐10b	52 primary PC and normal adjacent tissues (24 early biochemical relapse and 22 no/ late biochemical relapse)	2.15 (1.02‐4.51)	0.044	34
miR‐23a	3 pairs of primary prostate cancer and adjacent non‐tumor tissues 20 paired of prostate cancer and adjacent non‐tumor tissues. 123 prostate cancer tissues	0.389 (0.249‐0.608)	<0.0001	41
miR‐23b	118 pairs of PC and control 27 BPH and 20 tumor samples 48 samples	6 (3‐13)	<0.002	43
miR‐27b	41 noncancerous tissues and 49 PC tissues	0.255 (0.069‐0.944)	0.0407	44
miR‐34b	148 LCM matched human tissue samples 27 BPH and 20 tumor samples	3.3 (1.3‐8.7)	<0.02	51
miR‐106b	28 non‐cancerous tissues, 99 primary tumors, and 14 distant metastases/recurrence.	2.7 (1.1‐7.3)	0.014	53
miR‐100	49 prostate cancer (28 men without and 21 with biochemical recurrence)	3.045 (1.200‐7.737)	0.019	27
miR‐133b	135 PC, 18 prostatic intraepithelial neoplasia (PIN), and 25 normals	1.775 (1.013‐3.108)	0.045	59
miR‐150	167 PC 4 pairs of PC and adjacent normal tissues	1.90 (1.21‐2.98)	0.005	63
miR‐191	49 prostate cancer (28 men without and 21 with biochemical recurrence)	2.642 (1.030‐6.780)	0.043	27
miR‐205	Study cohort: 105 HRPC, 10 BHP validation cohort:78 HRPCa	Study cohort: 2.01(0.83‐4.85) Validation cohort: 0.82 (0.39‐1.7)	0.596	71
miR‐221	28 recurrent and 37 non‐recurrent prostate cancer cases	0.71 (0.32‐1.61)	0.42	28
miR‐222	28 recurrent and 37 non‐recurrent prostate cancer cases	0.51 (0.22‐1.18)	0.12	28
miR‐224	4 and 20 pairs of primary PC and adjacent non‐tumor frozen samples TMA: 114 PC tissues respectively from Caucasian and African‐American PC patients	0.31 (0.11‐0.86)	0.017	78
miR‐301a	585 prostate cancer	1.42 (1.06‐1.90)	0.002	81
miR‐383	TCGA database: 187 primary PC validation cohort: 112 PC FFPE tissues and matched adjacent normals	TCGA database: 0.661 Validation cohort: 0.897	0.0655	84
miR‐449b	36 PC 163 radical prostatectomy patients 40 patients (20 recurrent and 20 non‐recurrent patients)	1.9	0.003	85
miR‐466	48 pairs of LCM tissue samples validation cohort: 56 PC	17 (5‐50)	0.02	86
miR‐663	127 prostate cancer and 10 benign prostatic hyperplasia (BPH)	2.924 (1.981‐4.316)	<0.001	91
miR‐708	40 PC and 8 normal 96 paired of PC and normal	6 (2.2‐16.4)	0.0138	92
miR‐1207‐3p	PC patients of 389 CA and 15 moAA	1.8 (0.8‐4.3)	<0.001	93
miR‐3622b	100 pairs of PC and adjacent normals	0.407	0.2	94
let‐7b	cohort A: 98 high‐risk PC Cohort B: 92 FFPE samples cohort C: 21 pairs of PC and adjacent benign tissue	0.36 (0.161‐0.823)	0.02	107

RFS, recurrence free survival; HR, hazard ratio; CI, confidence interval; Refs, reference; PC, prostate cancer; BPH, benign prostate hyperplasia; CRPC, castration resistant prostate cancer.

**Table 4:** The overall survival of miRNAs in enrolled studies

miRNA	Samples	OS HR/RR (95%CI)	*P* value	Refs
miR‐23a	3 pairs of primary prostate cancer and adjacent non‐tumor tissues 20 paired of prostate cancer and adjacent non‐tumor tissues. 123 prostate cancer tissues	0.389 (0.249‐0.608)	<0.001	41
miR‐23b	118 pairs of PCs and controls 27 BPH and 20 tumor samples 48 samples	3.3 (4‐19)	<0.0001	43
miR‐132	Phase 1 cohort: 97 patients Phase 2 cohort: 89 patients	1.9 (1.1‐3.4)	0.02	29
miR‐150	167 PC 4 pairs of PC and adjacent normal tissues	1.87 (1.19‐2.94)	0.006	63
miR‐200a	Phase 1 cohort: 97 patients Phase 2 cohort: 89 patients	2.1 (1.2‐3.6)	0.009	29
miR‐200b	Phase 1 cohort: 97 patients Phase 2 cohort: 89 patients	3.8 (2.0‐6.9)	0.000006	29
miR‐200c	Phase 1 cohort: 97 patients Phase 2 cohort: 89 patients	3.8 (2.0‐6.9)	0.005	29
miR‐205	Study cohort: 105 HRPC, 10 BPH validation cohort: 78 HRPC	Study cohort: 2.04 Validation cohort: 3.1	0.0817	71
miR‐221	cohort 1: 134 PC cohort 2: 89 PC	cohort 1: 0 cohort 2: 0.029	<0.0001	74
miR‐224	4 and 20 pairs of primary PC and adjacent non‐tumor Taylor dataset: 149 primary PC tissues and 29 adjacent non‐cancerous prostate tissues	0.73 (0.31‐1.72)	0.046	76
miR‐429	Phase 1 cohort: 97 patients Phase 2 cohort: 89 patients	3.3 (1.8‐6.0)	0.00005	29
miR‐708	40 PC and 8 normal 96 pairs of PC and normal	6 (2.2‐16.4)	0.0223	92
miR‐1207‐3p	PC patients of 389 CA and 15 AA	1.8 (0.8‐4.3)	0.062	93
miR‐1290	23 CRPC patients 100 CRPC	1.79 (1.30‐2.48)	<0.004	30

OS,overall survival; HR, hazard ratio; CI, confidence interval; Refs, reference; PC, prostate cancer; BPH, benign prostate hyperplasia; CRPC, castration resistant prostate cancer.

### 3.2 miRNAs and PC diagnosis

miRNAs may regulate the wide range of biologic processes, and their deregulation are associated with PC onset, progression, and metastasis. More and more studies investigated differentially expressed miRNA as PC diagnostic and prognostic markers by comparing the expression levels of miRNAs in tumor tissues to that in BPH or normal controls. But there were the high variability in the data obtained from the different records. These could be caused by several factors as follows: (i) different sample groups; (ii) different detecting and verifying methods; (iii) small sample size. Nonetheless, these studies depicted a starting point, and some of the included records screened the same miRNA which was found with the same trend in multiple studies with different methods, as shown in Table 1. However, a confirmed diagnostic miRNA which could be translated into the clinic was not arised. Further confirmed experiments are needed in additional large patient cohorts.

In Figure 2, 22 miRNAs were reported to be consistently deregulated in different records. Among them, 6 miRNAs (miR‐34a, miR‐106b, miR‐183, miR‐200a/b, and miR‐301a) were up‐regulated in PC, while 16 miRNAs (miR‐1, miR‐23b, miR‐27b, miR‐34b/c, miR‐99b, miR‐125b, miR‐152, miR‐187, miR‐199a, miR‐204, miR‐205, miR‐224, miR‐452, miR‐454, and miR‐505) were down‐regulated. miR‐125b, miR‐205, miR‐1, and miR‐23b were the most commonly detected to evaluate their diagnostic efficacy between PC patients and non‐cancerous individuals. In the studies about the most obviously up‐regulated miR‐200a and miR‐200b, the pooled expression values were 5.17 (95%CI 3.22‐7.13) and 4.08 (95%CI 2.91‐5.24), respectively (Figures 2N and 2O). While miR‐199a was most significantly down‐regulated, which pooled value was −4.23 (95%CI −16.22, 7.76) (Figure 2M). More potential biomarkers were summarized in Table 2, 134 PC related miRNAs were listed which had the diagnostic potential to be aberrantly expressed in PC patients compared with healthy controls. A 60 up‐regulated miRNAs and 63 down‐regulated miRNAs were able to discriminate PC patients from BPH or healthy individuals. The remaining 11 miRNAs were not statistically significant in the studies. Any miRNA‐based clinical screening still lacks a consensus signature to be applied in the routine assay, and needs further validation in an intended use population.

### 3.3 Publication bias and subgroup analysis

The high heterogeneity between the data from the included records could be associated with several factors: different study design, different races of patients, different methods of sample collection and detection, incomplete information, and small sample size. There were also many difficultly statistical factors: proportion of contaminating cells, limited tumor size and differences in miRNAs stability and processing. In addition, different control samples (BPH or adjacent normal or unmatched normal) and the different characteristics of PC (low/high‐risk or metastasis or recurrence) could explain, at least in part, the different results. Significant heterogeneities (*P *< 0.05, *I* ^2 ^> 50%) were found in most miRNAs expression profiles, we performed the subgroup analyses to seek the source of heterogeneity, which include ethnicity, sources of control (BPH or N), and sample types (serum/plasma or urine), etc.

To assess publication bias of 11 studies on miR‐21, the funnel plot was drawed. As shown in the Figure 3C, significant publication bias was found in the pooled analysis of miR‐21 (*P *< 0.00001, *I* ^2^ = 95%), most of the research data was distributed on the edge line. In order to avoid the effect of heterogeneity, we performed four subgroup analyses divided by ethnicity, and sample categories, including: China, Brazil, local versus meta, and PC versus control. Unfortunately, the heterogeneities were significantly reduced in Brazil subgroup and local versus meta subgroup, while the expression of miR‐21 in other subgroups still had obvious heterogeneity. In Brazil subgroup, I2 value was less than 50%, but SD value from the first study by Betina Katz^15^was too large, and covered the scope of the other two data. Therefore, we believe that the study on miR‐21 still needs to be further expanded. We did not find corresponding increased miR‐21 in Chinese by merging four studies^16^, ^17^, ^18^, ^19^ (Figure 3C). When stratified by the category of detected samples, increased expression of miR‐21 showed consistency in local versus meta subgroup, but no statistically significant result was observed in PC versus control subgroup.

Five studies on miR‐100 had obvious heterogeneity, as shown in the Figure 3F. After carefully reviewing the five full‐texts, they were divided into three subgroups, including: Brazil, urine and recurrence and non‐recurrence. Among them, heterogeneity in the Brazil subgroup was significantly reduced (*P* = 0.24, *I* ^2^ = 26%). Subgroup analysis of miR‐141 expression showed that miR‐141 expression was consistently up‐regulated in four studies of PC versus control subgroup and more obviously up‐regulated in serum samples data from Heather H. Cheng.^20^ Four studies on miR‐200c had also obvious heterogeneity, as shown in the Figure 3K. Three subgroups: PC versus control, serum and urine were classified according to different sample characteristics. Fan feng et al^21^ collected the serum samples from 50 PC patients and 10 normal controls, while Heather H, Cheng et al^20^ detected the miR‐200c expression levels in patients' urine samples. The heterogeneity of miR‐200c expression in PC versus control subgroup significantly reduced (*P* = 0.98, I2 = 0%). Six studies on miR‐221 were divided into four subgroups: local versus meta, aggressive versus non‐aggressive, PC versus control and urine, and the heterogeneity in PC versus control subgroup was significantly reduced to 0%. In addition, existing data showed that the expression of miR‐221 in primary PC was less than that in normal tissues, but miR‐221 was significantly increased when PC progressed to more malignant stages (metastasis or recurrence or hormone resistance). Among them, Tong's research data were divided into two parts, which were included in local versus meta subgroup and PC versus control subgroup, respectively. The studies on miR‐15a and miR‐16 were divided into two subgroups: PC versus meta, PC versus control. Results showed that both of miR‐15a and miR‐16 were up‐regulated in metastasis PC, while their expression levels were lower in PC tissues than in non‐cancerous tissues. Inconsistently expression of let‐7c was reported. The three studies on let‐7c were divided into two subgroups: high‐risk versus meta and high‐risk versus control, and the research data from Katia R. M. Leite 2013^22^ were separately counted in the two subgroups because two sets of data were involved. The heterogeneity was significantly reduced to 0% and 12%. The study of miR‐143, 145 191, −25‐32 was divided into two subgroups, PC versus meta, PC versus control. Moreover, miR‐222 and miR‐375 were inconsistently expressed in prostate tumor tissues and matched normal tissues (Figures 3M and 3N). So it was essential to conduct subgroup analyses on miR‐222 and miR‐375 expression. Five studies on miR‐222 could be divided into three subgroups: PC versus control, China, and urine. The study of D Lin was a comparative study on the malignant and non‐malignant PC in China. The heterogeneity in PC versus control subgroup significantly decreased to 27%. Five studies on miR‐375 were divided into three subgroups: PC versus control, serum, and urine. The heterogeneities in PC versus control and serum subgroups were significantly reduced to 23% and 0%, respectively. The analyses of the above‐mentioned subgroups showed that the expression of miR‐375 in the urine samples were widely different, and also deviated from the expression profiles of tissues and plasma samples.

In addition to the above mentioned miRNAs expression data, there were also significant heterogeneities in the studies on seven miRNAs (Figure 4). Among them, studies on miR‐10b, miR‐18a, miR‐30c, and miR‐206, research data from Beatriz A. Walter^23^ deviated significantly from other research data. The heterogeneity decreased significantly when we rejected the deviant data. Moreover, in several studies on miR‐139‐5p and miR‐182, Cheng Pang^24^ and Fan Feng^21^ detected miRNAs expression profiles in whole blood and urine, respectively, which could explain the causes of heterogeneity. Finally, in three studies about miR‐146 a, Bin Xu^25^ collected ADPC and AIPC patients' samples in China, which were obviously different from the other two studies by Katia R. M. Leite^26^, ^27^ in Brazil.

Studies on 14 miRNAs (miR‐31, miR‐124, miR‐125a, miR‐133a/b, miR‐154, miR‐181a/b/c, miR‐203, miR‐210, miR‐218, miR‐378, and miR‐548c) had separately 2‐3 studies with significant heterogeneity (Figure 5). These studies only opened the gateway for the diagnosis and prognosis potential of 14 miRNAs, more researches are needed to confirm their application value in clinic.

### 3.4 miRNA expression and recurrence‐free survival

Biochemical recurrence (BCR) was considered as the first key point to estimate treatment success after RP. BCR can predate the development of metastases and other signs of clinical progression, or ultimately death. Recently, a lot of studies attempted to find miRNAs to be potential predictors for patients with biochemical failure. We summarized previous data in the meta‐analysis, miR‐30c, miR‐129, miR‐145, and let‐7c were found to have the same trend to predict BCR in eight articles (Figure 6B‐E). While the relationship of miR‐21 and BCR were studied in four articles with significant heterogeneity (Figure 6A). After reviewing the four full texts, we found that Ernest K Amankwah^28^ examined the effect of the interaction between obesity and miR‐21 expression on PC recurrence. Obese patients were included in the study. Removing the data from Ernest K Amankwah, miR‐21 could distinguish biochemical failure patients from non‐recurrence (Figure 6A).

In remaining 20 articles, we found prostate tumors with high levels of miR‐10b, miR‐100, miR‐106b, miR‐133b, miR‐150, miR‐191, miR‐301a, miR‐449b, miR‐663, or miR‐1207‐3p have significant decrease in RFS, while low levels of miR‐23a/b, miR‐27b, miR‐34b, miR‐224, miR‐466, miR‐709, and let‐7b were significantly correlated with poorer RFS (Table 3). Five miRNAs (miR‐205, miR‐221, miR‐222, miR‐383, and miR‐3622b) were detected no correlation between the expression levels and tumor progression (*P* > 0.05).

### 3.5 miRNA expression and overall survival

A total of 11 records comprised OS analysis involving 15 miRNAs (Table 4 and Figure 6F). Among three articles on miR‐375, significant heterogeneity was observed (*P* = 0.03, *I* ^2 ^= 70%). After reviewing three full texts, we found plasma samples were used in the studies of Hui‐ming Lin^29^ and Xiaoyi Huang,^30^ while serum samples were used in the study of Sven Wach.^31^ Removing the data from Sven Wach, the heterogeneity was markedly decreased (*P* = 0.67, *I* ^2^ = 0%) (Figure 6F). Hence, a fixed model was applied to calculate a pooled RR and 95%CI, and we found that patients with high miR‐375 expression had significantly poorer OS compared to low miR‐375 expression (RR = 2.93, 95%CI, 1.96‐4.40) (Figure 6F).

In the other eight studies involving 14 miRNAs (Table 4), eight miRNAs (miR‐132, miR‐150, miR‐200a/b/c, miR‐429, miR‐708, and miR‐1290) were showed that increased expression predicted significantly worse OS, and low expression of four miRNAs (miR‐23a, miR‐23b, miR‐221, and miR‐224) were associated with poorer OS. Moreover, in the analyses on miR‐205 and miR‐1207‐3p, no statistically significant results were observed. It was worth noting that miR‐200a, miR‐200b, miR‐200c, and miR‐429 were the members of the same family, their change trends were consistent in different studies, and all of them were associated with poorer OS.

## 4 DISCUSSION

The major challenges for PC clinical management were its accurate diagnosis and dynamic monitoring after RP, chemotherapy or radiotherapy, etc. Although PSA routinely screening improved the ratio of early detection, its levels was poorly associated with tumor aggressiveness, and had a little help to predict PC patients' prognosis. Moreover, biopsies were not only invasive but also not conclusive, for example, sampling errors could lead to missed diagnosis and wrong therapies in clinic, especially in the cases with multifocal PC.

Recently, miRNAs had been found to be closely associated with a variety of tumors by regulating their target genes to affect carcinogenesis and progression. And a number of researches showed a significant correlation between the expression levels of miRNAs and the diagnosis and prognosis of PC. These study data would be helpful miRNAs as biomarkers to be transfer into the clinical application for diagnosis and prognosis of PC. Moreover, Compared to mRNAs, clinical samples containing miRNAs are more likely to be collected and detested because miRNAs are stable not to be easily degraded. The expression profiles of miRNAs are special in various cancer or normal tissues. And they can be accurately quantified by microarray, qRT‐PCR, and RNA sequencing in not only frozen or fresh or formalin‐fixed paraffin‐embedded tissues, but also serum or plasma samples, even in urine or saliva samples. However, these results on the clinical value of miRNAs were inconsistent and even contradictory due to the clinical complexity of PC. Therefore, it is necessary to conduct stratified and systematic analyses to confirm their expression pattern and application scope.

By meta‐analyses of included studies, we successfully come to some valuable conclusions for future applications in clinic. The most studied miRNA was miR‐21, with 11 articles providing the data of its expression level in clinical PC samples. Secondly, the expression profile data of miR‐221 and miR‐205 were clearly reported in seven and six studies, respectively. And the expression levels of 7 miRNAs (miR‐25, miR‐32, miR‐100, miR‐125b, miR‐141, miR‐222, miR‐375) were reported in five literatures. In addition, the most obviously increased miRNAs were the members of the miR‐200 family: miR‐200a and miR‐200b, their HR and 95%CI were 5.17 (3.22‐7.13) and 4.08 (2.91‐5.24), respectively. The most significantly decreased miRNA was miR‐199a, its pooled HR and 95%CI was −4.23 (−16.22‐7.76).

In order to remove the interference of genetic backgrounds due to patients' ethnic groups, the included studies were classified into China subgroup and Brazil subgroup, etc. We found increased miR‐21 expression could distinguish PC patients from normal controls, and could predict a significantly poor RFS. The expression of miR‐100 in the Brazilian population was significantly reduced, and HR and 95%CI was −77.57 (−110.47, −44.67). The different expression levels and predictive values of miRNAs may be explained by the differences of hereditary backgrounds and environmental exposures.

Second, we conducted subgroup analyses depending on the pathological types of PC to classify the enrolled studies into subgroups of cancer categories: normal controls/BPH, primary/local PC, metastatic PC, high‐risk PC, and recurrence PC/non‐recurrence PC subgroups, etc. In the comparisons of the expression profiles of miRNAs in primary/local PC versus metastatic PC subgroups, we found that miR‐21 and miR‐32 were up‐regulated in metastatic PC tissues, while miR‐25, miR‐143, miR‐145, miR‐191, and let‐7c were down‐regulated. In subgroup analyses of PC versus control, we found that miR‐141, miR‐200c, and miR‐375 were increased, while miR‐30 c, miR‐143, miR‐145, miR‐191, miR‐221, miR‐222, and let‐7c were reduced. Among them, low expression of three miRNAs (miR‐30c, miR‐45, and let‐7c) predicted worse RFS, the HR 95%CI were 0.32 (0.15‐0.66), 3.86 (1.85‐8.03), and 3.14 (1.49‐6.60), respectively. In addition, the expression model of miR‐15a and miR‐16 was special, both of them were lower expressed in PC tissues than that in normal controls, and their expression levels were increased again when PC progressed to malignant metastatic stages.

Finally, we performed subgroup analyses to clarify the diagnostic values of miRNAs based on the data of serum/plasma and urine samples, etc. We found that high‐expression of miR‐375 was significantly associated with a worse OS (HR = 2.93, 95%CI 1.96‐4.40) in serum/plasma subgroup, and its high‐expression was also shown in tissue subgroup (HR = 7.41, 95%CI 6.49‐8.33) and urine subgroup (HR = 1799.29, 95%CI 1796.45‐1802.13). In addition, we processed subgroup analyses of the expression levels of other five miRNAs in serum or urine samples. Among them, the members of miR‐200 family: miR‐141 and miR‐200c were up‐regulated, while the expression levels of miR‐221 and miR‐221 were decreased. The expression of miR‐100 was increased in the urine samples, which was contrary to its expression in patients' tissues. Although the detection of miRNAs in tissues was widely accepted by researchers and doctors to diagnose and predict PC progression, the detection in serum or urine samples was more convenient and uninjurious, which could dynamically monitor the therapeutic effects and patients' prognosis at any time point of the lifetime of PC patients.

The meta‐analysis has some merits. First, we strictly followed the literature inclusion criteria and the quality of enrolled literatures was satisfactory. Second, we conducted subgroup analyses to effectively minimize the influence of heterogeneity among the enrolled studies, and to further explore the scope of application for miRNAs as a prognostic biomarker of malignant tumors. All of these have increased the statistical power of the meta‐analysis. But there are also many shortcomings in the meta‐analysis. First, only a few articles are eligible for a kind of miRNA leading to the relative shortage in subgroup analyses. Secondly, after data integration and subgroup analyses, some miRNAs data still lack statistical significance, such as miR‐15a (*P* = 0.45), miR‐16 (*P* = 0.69), miR‐21 (*P* = 0.49), miR‐25 (*P* = 0.83), miR‐191 (*P* = 0.49), and miR‐200c (*P* = 0.63), etc. Besides, no study is carried out in Africa, which blocks the integrated investigation of the association between miRNAs expression and PC diagnosis and prognosis. Finally, because of the lack of unified cut‐off value of miRNAs expression in different researches, which would reduce the potency of miRNAs as predictive biomarkers. Therefore, the application value of miRNAs as prognostic factors for PC is still controversial, requiring more researches to verify.

## 5 CONCLUSIONS

The potential use of miRNAs as diagnosis and prognosis factors for PC in the clinic was based on a growing body of investigations in the last decades. Currently, ongoing researches were still controversial that delayed the transformation from bench to bedside. Nevertheless, the potential value of miRNAs used in clinical practice had been generally accepted, which represented not only promising biomarkers for PC but also candidated therapeutic targets. Besides, detecting the expression levels of miRNAs in serum or plasma or urine samples was more exciting than detecting miRNAs in tissues, because of low cost, rapid test, and noninvasion, etc. However, in this meta‐analysis, we found that the expression profiles of miRNAs in the blood samples were different from that of the tissues, and the deviation in the urine samples was more obvious.

Due to the lack of relevant data, further studies in larger sample sizes are needed to conduct more precise stratification between miRNAs expression levels and different progression stages of PC. We will also continue to evaluate and report the clinical value of miRNAs detection when larger studies further verify the validity of miRNAs. The guidelines on study design and sample collection still need to be further improved, in particular, the detecting platforms should be clearly defined. Taken together, the meta‐analysis underline that the use of miRNAs as biomarkers for diagnosis and prediction of PC is promising, though not yet a reality in clinical practice.
